# Critical patch size generated by Allee effect in gypsy moth, *Lymantria dispar* (L.)

**DOI:** 10.1111/j.1461-0248.2010.01569.x

**Published:** 2011-02

**Authors:** E Vercken, A M Kramer, P C Tobin, J M Drake

**Affiliations:** 1 UMR IBSV, Institut National de la Recherche Agronomique (INRA)400 Route des Chappes, BP 167, 06903 Sophia-Antipolis Cedex, France; 2 Odum School of Ecology, University of GeorgiaAthens, GA 30605, USA; 3 Forest Service, United States Department of AgricultureNorthern Research Station, 180 Canfield Street, Morgantown, WV 26505, USA

**Keywords:** Allee effect, critical area, dispersal, extinction, generalized additive models, gypsy moth, invasion, reaction-diffusion, Voronoi tessellation

## Abstract

Allee effects are important dynamical mechanisms in small-density populations in which per capita population growth rate increases with density. When positive density dependence is sufficiently severe (a ‘strong’ Allee effect), a critical density arises below which populations do not persist. For spatially distributed populations subject to dispersal, theory predicts that the occupied area also exhibits a critical threshold for population persistence, but this result has not been confirmed in nature. We tested this prediction in patterns of population persistence across the invasion front of the European gypsy moth (*Lymantria dispar*) in the United States in data collected between 1996 and 2008. Our analysis consistently provided evidence for effects of both population area and density on persistence, as predicted by the general theory, and confirmed here using a mechanistic model developed for the gypsy moth system. We believe this study to be the first empirical documentation of critical patch size induced by an Allee effect.

## Introduction

Allee effects are a small-population phenomenon in which individual fitness increases with population density (Allee 1931). Allee effects are central to many fundamental problems in population biology including the evolution of mating systems (Gascoigne *et al.* 2009; Leducq *et al.* 2010), density-dependent selection (Asmussen 1979) and the biogeography of isolated populations (Kramer *et al.* 2008) and to ecological applications as diverse as forecasting spread of invasive species (Veit & Lewis 1996; Taylor & Hastings 2005), assessing viability of threatened populations (Wittmer *et al.* 2010) and setting harvest quotas for exploited populations (Berec *et al.* 2007). Allee effects have nevertheless been exceedingly poorly documented (Fowler & Baker 1991; Kramer *et al.* 2009), primarily because directly measuring fitness in nature is complicated as organisms in low-density populations are difficult to locate (Courchamp *et al.* 2008).

There are many causes of an Allee effect at the level of individual organisms, including mate-finding failure, lack of predator satiation or avoidance and reduced foraging efficiency (Berec *et al.* 2007). At the population level, however, Allee effects are always expressed as an increase in population growth rate with increased population size caused by positive demographic feedbacks (Courchamp *et al.* 2008). The consequences of these feedbacks in locally well-mixed populations are theoretically well understood and include the creation of an interior critical point (an unstable equilibrium, the ‘Allee threshold’) and the associated bistability (extinction or persistence at a density greater than the Allee threshold) characteristic of strong Allee effects (Wang & Kot 2001). In contrast, Allee effects in spatially distributed populations are relatively poorly understood (but see Robinet *et al.* 2008). Documented effects are primarily revealed as colonization patterns (Soboleva *et al.* 2003; Davis *et al.* 2004) or spread rates (Keitt *et al.* 2001; Johnson *et al.* 2006; Tobin *et al.* 2007).

Here, we report the first detection of Allee effects by means of another spatial phenomenon, a critical area, previously predicted by theory but not observed empirically. This criticality is different from the critical density induced by strong Allee effects in non-spatial models and arises from the interaction between the geometry of spread and positive density dependence at the front of an expanding population. Specifically, because of an Allee effect, change in total population size is determined by a race between reproduction in the population core (where the local density exceeds the Allee threshold) and diffusion at the periphery (where individuals do not contribute to growth because they are below the Allee threshold). As the radius *r* of the occupied area tends to small values, the ratio of periphery to core area, 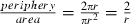 for a circular patch, increases and diffusion dominates, leading to extirpation of the incipient population. In contrast, as the occupied area increases, the ratio of periphery to core tends to zero so that population dynamics are dominated by the core and expansion occurs. It follows that diffusion on the periphery and growth in the core are balanced at an intermediate radius, which defines the critical area that must be occupied for growth to occur. A population with size greater than the classical critical density is therefore a necessary, but not sufficient, condition for growth of a population with an Allee effect in space.

To our knowledge, this phenomenon was first predicted by Lewis & Kareiva (1993), who derived and solved a partial differential equation (PDE) model for growth and dispersal in continuous time of a population with Allee effects. This prediction has since been shown to be a general property of Allee effects in spatial models including other PDE models (Soboleva *et al.* 2003), integrodifference equation models (Kot *et al.* 1996) and individual-based simulations (Etienne *et al.* 2002). However, none of these studies provided empirical evidence of critical areas.

We sought to detect this phenomenon by investigating the geometry of incipient patches of the European gypsy moth, *Lymantria dispar* (L.) (Lepidoptera: Lymantriidae), in the United States. This system is an ideal one in which to search for a critical effect because extensive spatio-temporal monitoring data are available (e.g. Tobin & Blackburn 2007) and gypsy moth population ecology is well documented (e.g. Elkinton & Liebhold 1990), which makes it possible to relate local population processes to patterns observed at large geographical scales. To document the fate of incipient populations, we first estimated the boundaries of occupied areas from point data. Our strategy involved the recursive identification of neighbouring cells in a Voronoi tessellation of the available presence/absence data. We applied this method to 12 years of male moth density data, collected from pheromone-baited traps deployed annually over the gypsy moth invasion front from Wisconsin to North Carolina. To validate the prediction of a critical invasion area, we analysed the relationship between population area and persistence from 1 year to the next using spatial nonparametric statistics. To verify that local processes such as mating and dispersal could cause such a relationship, we developed a mechanistic model of gypsy moth population growth rate. To understand the sensitivity of our detection procedure, we explored the influence of the interaction between dispersal distance and population area on emigration rate and population growth in this model. These analyses provide the first empirical support for the prediction of a critical area for invading populations subject to an Allee effect.

## Methods

### Study system

The European gypsy moth is native to most of temperate Eurasia and was introduced to North America outside of Boston, MA, in 1869 (Liebhold *et al.* 1989). Its current range extends from Ontario to North Carolina and Nova Scotia to Wisconsin (Tobin *et al.* 2009). The gypsy moth is univoltine. Overwintering eggs hatch in the spring, and larvae feed on the foliage of more than 300 tree species (Elkinton & Liebhold 1990). Dispersal is primarily at two scales: larval ballooning occurs at characteristic distances of hundreds of metres (Mason & McManus 1981) and anthropogenic movement occurs at distances of up to hundreds of kilometres (Hajek & Tobin 2009). Adults emerge in mid- to late-summer, and live only a few days (Sharov *et al.* 1995). European gypsy moth females are unable to fly, and males rely on pheromone signals to locate mates (Elkinton & Liebhold 1990). The effectiveness of this mate-finding system is known to decrease quickly with distance, inducing a strong Allee effect (Robinet *et al.* 2008; Tobin *et al.* 2009). The magnitude of this Allee effect varies geographically because of different rates of male moth dispersal (Tobin & Blackburn 2008) resulting in differing male moth densities and altered female mating success across the species’ introduced range (Sharov *et al.* 1995; Contarini *et al.* 2009). This spatially varying Allee effect therefore results in regional differences in establishment success and speed of spread in the United States (Whitmire & Tobin 2006; Tobin *et al.* 2007).

**Figure 1:**
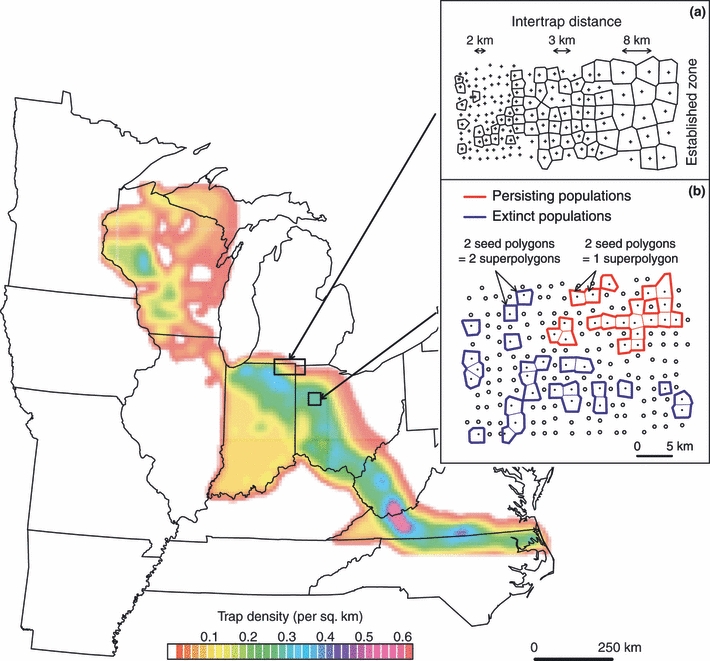
Map of the distribution of pheromone-baited traps along the gypsy moth invasion front, 2003. (a) Variation in intertrap distance across the transition zone between established and unestablished areas. (b) Construction of super-polygons: empty traps (white circles) are not included in polygons; seed polygons that share a common edge are merged into a super-polygon.

Along the leading edge of the gypsy moth distribution, new populations are monitored over a ≈170 km wide band from Wisconsin to North Carolina under the Slow-the-Spread (STS) program (Fig. 1; Tobin & Blackburn 2007). Within this transition zone, which separates areas of well-established populations that undergo periodic outbreaks (Liebhold & Elkinton 1989) from areas in which gypsy moth is absent, over 100 000 pheromone-baited traps were deployed each year during the later stages of the pilot (1996–1999) and formal STS programs (2000–present). Traps, which are specific to gypsy moth males, are deployed from 500 m to 8 km apart, depending on background population densities. Within the portion of this transition zone where populations are usually at very low densities, traps are typically set 2 km apart or less, which has been shown to be sufficient to detect low-density isolated colonies (Sharov *et al.* 1998). The data analysed here are annual trap catches from Wisconsin to North Carolina during 1996–2008. As some areas within this transition zone are treated to eliminate gypsy moth populations, traps within 1.5 km from a treated area were excluded from our analysis (on average, < 2% of the transition zone was treated with pesticides each year; Tobin & Blackburn 2007).

### Population boundaries and persistence

Trap data were used to define the spatial extent of isolated gypsy moth populations as territories inside which the gypsy moth was present, defined by the capture of at least one male moth in every trap, surrounded by areas in which the gypsy moth was absent from all traps. We estimated population boundaries by first determining the spatial extent represented by each trap and then identifying groups of adjoining gypsy moth-positive traps as follows. The areal unit sampled by trap *i* was defined to be the polygon containing all points closer in Euclidean distance to *i* than to any other trap, a definition that associates trap locations (a point process) with a Voronoi tessellation of the naturally continuous space in which the point process is embedded (Fig. 1; de Berg *et al.* 2008). The Voronoi diagram for each year was calculated with the package ‘tripack’ (v. 1.3-3; Gebhardt 2009) in R (R Development Core Team 2008) and resulted in a set of polygons, which we refer to as ‘seed polygons’ (Fig. 1), equal in number to the total quantity of traps. Artefacts at the boundaries of the trapping range were removed prior to analysis. A recursive algorithm was used to merge adjacent seed polygons into contiguous occupied regions. The algorithm proceeded by iteratively considering each seed polygon. If the focal polygon was occupied, each of its neighbours was inspected. Any neighbour which contained a gypsy moth-positive trap was joined with the seed polygon along their adjacent edge. This process was repeated using the new (joined) polygon as a new seed. This iterative process resulted in the largest group of adjacent polygons in which all members were positive for gypsy moth presence. Sets of adjacent polygons were merged (Fig. 1b), resulting in a ‘super-polygon’, which we consider to represent a single incipient population.

Persistence was assessed at the population (i.e. super-polygon) scale. Population persistence was scored as a binary variable accounting for the presence of gypsy moth males in traps in year *t* + 1 within the boundaries of a super-polygon from year *t* (0 when no moths were recorded in traps in year *t +*1; 1 when at least one trap recorded at least one moth). Relative population density was estimated by summing counts of male moths captured in all traps in a super-polygon, divided by the number of traps. Super-polygons larger than 10 000 km^2^ or in which a trap was not present in the following year were excluded from subsequent analyses.

### Evidence for critical area in natural populations

Hypothesized effects of area, density and other spatial covariates (elevation, frost index and preferred host density; see Appendix S1 in Supporting Information) on population persistence were tested using geoadditive models (Kammann & Wand 2003). Geoadditive models are generalized additive models that fit smoothing splines to nonlinear relationships between variables including spatial covariates (Wood 2006), and are appropriate for the analysis of spatial data (Beale *et al.* 2010). A previous analysis, also based on STS trap catch data, revealed regional (as defined by political boundaries) variation in the strength of the Allee effect with a consequent effect on gypsy moth spread rates; however, the relationship between invasion speed and Allee effect strength did not vary along either a latitudinal or longitudinal gradient (Tobin *et al.* 2007). By specifying a generalized additive model with more degrees of freedom, we aimed to incorporate these regional differences as well as variation in important covariates such as climate, weather, elevation and differences in host tree presence and density. Two additional factors that vary with location and probably affect observed gypsy moth dynamics are trap density (the power of detection is higher in areas with higher trap density) and distance from established gypsy moth range (populations close to the established zone are more likely to receive individuals from established populations; Tobin & Blackburn 2007). By allowing the effect of location to vary nonlinearly, we hoped to improve model accuracy and more fully separate the effects of area and density on persistence.

Persistence in each of 12 pairs of consecutive years was modelled as a binary response with a logit link function and binomial response distribution using the package ‘mgcv’ (v. 1.5-5; Wood 2009) in R (R Development Core Team 2008). Geographic location was fit with a smooth term (thin-plate regression spline) on the *x* and *y* coordinates of the polygon centroid with maximum degrees of freedom set to 100 and the model degrees of freedom multiplied by γ = 1.4 to reduce over-fitting (Wood 2006). The estimated degrees of freedom (smoothness) were determined automatically using Wood’s (2006) unbiased risk estimator. The effect of population area on persistence was modelled as the log_10_-transformed area of the super-polygons. Critical area was conventionally defined as the area for which 50% of the populations went extinct. Models were evaluated using covariate *P*-values, deviance explained and AIC.

As gypsy moth invasion dynamics are already known to present Allee effects (Tobin *et al.* 2009), population density was expected to be an important covariate of persistence. In the data analysed here, density (as defined above, i.e. average male count per trap) was significantly positively correlated with area in all years because most very large populations are found closest to the established area, where densities are higher. We therefore defined ‘residual density’ as the residuals of the fit linear relationship between population density and area in each year. Residual density provides a relative measure of density independent of area in which positive values reflect populations with more individuals than expected for a given area, whereas negative values reflect populations with fewer individuals than expected. A positive influence of these residuals on population persistence therefore represents a positive effect of population density independent of area effects, which is expected for colonizing populations subject to an Allee effect.

A weaker prediction of the theory is that populations occupying an area smaller than the critical area will shrink even if they do not go extinct in the next time step. To confirm that the change in population area in years *t* and *t* + 1 followed this pattern, population area in year *t* + 1 was estimated and the change in area plotted with the predicted critical area for persistence (Appendix S2). A result consistent with this prediction would provide additional evidence that population persistence depends on the area occupied.

### Effect of area in model populations

Only strong Allee effects (Allee effects that give rise to a critical local density) are expected to induce a critical area (Lewis & Kareiva 1993). To confirm that intrinsic processes of gypsy moth population dynamics could cause the patterns we observed, we explored the interaction between an Allee effect and random dispersal in a mechanistic model of population growth in one generation. In this model, the multiplicative growth rate λ is given by

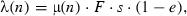
(1)

where μ is the probability of mating (an increasing function of density *n*), *F* is the number of female eggs produced by each mated female, *s* is the survival rate to maturity and *e* is the emigration rate. Using parameter values corresponding to published data on gypsy moth biology (Robinet *et al.* 2008), this model generates a strong Allee effect [λ(*n*) < 1 at some density *n* > 0] because the probability of mate-finding decreases very quickly when with the distance between individuals increases (Figure S5). Additional model details and results are given in Appendix S3.

## Results

### Evidence for critical area in natural populations

In each year, between 7965 and 22 517 seed polygons (mean = 16 016, SD = 4505), and between 1450 and 2353 super-polygons (mean = 1830, SD = 248) were constructed (see Table S1 for details). After removing super-polygons that did not include any traps in the subsequent year and anomalous cases at the boundaries of the invaded area, there were between 572 and 1885 super-polygons remaining for analysis for every year pair (mean = 1235, SD = 314). The distribution of super-polygon areas and residual densities (see below) was homogeneous between years (Figure S1).

Analysis with geoadditive models provided strong evidence for the importance of population area, which was further supported by the graphical analysis of the rate of area change (Appendix S1; Figure S4). In the geoadditive models, persistence was positively dependent on area in every year and on residual density in 10 of 12 years (Table 1). In 6 of 12 years, the interaction between area and residual density was significantly negative (*P*< 0.05). This interaction was driven by the lowest density populations which had negative residual densities and was not present when these were excluded, indicating the effect of area was enhanced in low-density populations, and not that persistence declined in populations with large area. The geographic smooth term explained the largest proportion of total variance and was highly significant (*P*< 0.0001) in all years. The model including residual density, area, residual density × area interaction and space explained 29–48% of the deviance in persistence and had AIC values lower than or equivalent to a model including frost index as a covariate. Models including preferred host density and elevation differed in sample size, complicating comparison by AIC, but showed no or little improvement in deviance explained in 8 of 12 years and minor, differing contributions in the remaining years (Figure S2; details on model selection are given in Appendix S1).

**Table 1:** Results of generalized additive model of persistence

	Coefficients				
			
Year	Log_10_ (area)	Density‡	Interaction	e.d.f. of smooth parameter§	% deviance explained
1996	1.42***	0.50	0.28	32.2***	39.7
1997	0.97***	3.02**	−1.04	20.0***	28.6
1998	1.39***	1.57†	−0.13	22.1***	47.8
1999	1.61***	2.19**	−0.64	40.7***	45.8
2000	2.43***	4.86***	−2.35**	36.3***	38.7
2001	2.59***	5.00***	−2.67***	24.0***	32.3
2002	1.65***	3.10***	−0.73	43.1***	35.9
2003	1.90***	4.86***	−1.87*	32.8***	36.1
2004	2.06***	4.01***	−2.04**	37.8***	29.7
2005	1.79***	1.14†	−0.79†	40.1***	35.2
2006	2.08***	2.33***	−1.09*	31.5***	38.5
2007	2.10***	−0.55	0.72	32.1***	37.5

Significance codes

† < 0.05

* < 0.01

** < 0.001

*** < 0.0001.

‡ Population density corrected for area (see Methods).

§ The estimated degrees of freedom for a smooth function of latitude and longitude. The significance code refers to the *P*-value on the null hypothesis of no effect of space.

**Figure 2:**
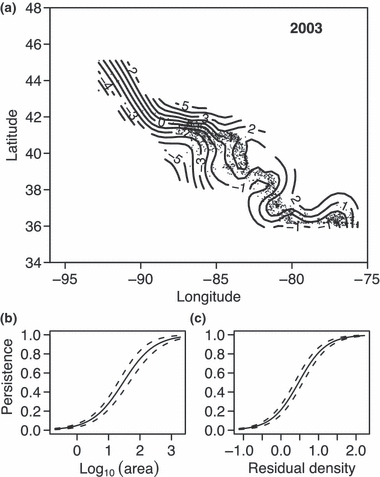
(a) Contour plot of the smoothing function for the effect of latitude and longitude on population persistence, shown in 2003 as an example. Contour labels represent standard deviations (positive is higher persistence, negative is lower persistence). Dots are the centroid of each super-polygon. (b, c). Partial dependence plots of the effect of area (b) and residual density (c) on population persistence in 2003. In these plots, the values of all predictors but the one on the *x*-axis are fixed at their median values to isolate the partial contribution of the *x*-axis predictor to the response variable. In (b), the area at which persistence = 0.5 is the predicted critical area at the median residual density and spatial location. Plots for all years are presented in Figure S3.

**Figure 3:**
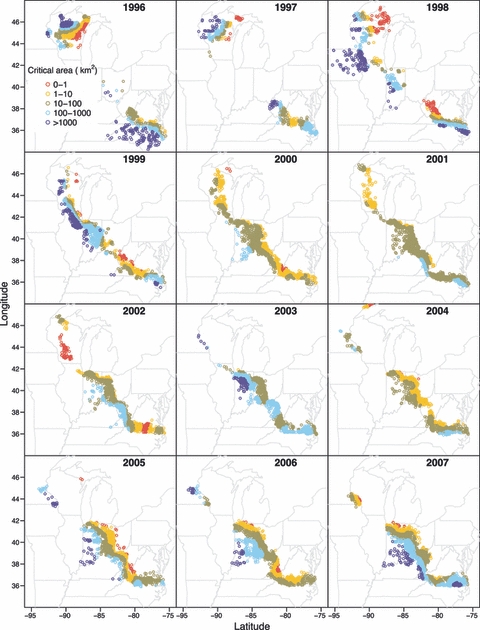
Estimated critical area for each population patch. Critical area was estimated at the centroid of each super-polygon (circles) as the area with a predicted persistence of 0.5 at the median residual density. Populations at the front of the expansion tend to have a larger critical area.

The model presented in Table 1 was therefore retained as the best model for year-to-year comparison and other models were not considered further. We note that in this model the geographic smooth term tended to follow the contour of the invasion front so that persistence declined with distance from the established range (Fig. 2a; Figure S3). Furthermore, partial effects of area and residual density (Fig. 2b,c showing the isolated effect of area or residual density on persistence when the other covariates are held constant at their median values) varied considerably from year to year (Figure S3). The partial effect of area provides a predicted critical area for a given year defined as the area at which persistence equals 0.5. However, because the median geographic coordinates, at which partial effects of area and residual density were estimated, are arbitrary and vary unpredictably, the estimated critical area for each trap location (at the median residual density) is more informative than a single estimate of critical area (Fig. 3). Lower persistence farther from the established range manifests as very high critical areas in these regions, while most positive traps occurred in locations with a critical area of 1–100 km^2^ (Fig. 3). This main finding is reinforced by analyses of the change in area between subsequent years (Appendix S2).

### Effect of area in model populations

Assuming random movement and a circular population, a simple geometric analysis of eqn 1 revealed that emigration rate was negatively correlated with population radius, and therefore population area (eqn 6, Appendix S3). For any given density above the Allee threshold, the model predicts the existence of a critical population radius, under which the population growth rate is < 1 and the population decreases. This critical radius decreases when population density increases (Figure S6a). The value of the critical radius also depends on the value of model parameters (fecundity, survival, dispersal distance); however, the qualitative behaviour of the system (i.e. the existence of a critical radius) is always conserved when parameters vary (Figure S6). The model results confirm for gypsy moths the general prediction of a critical area in the presence of an Allee effect.

## Discussion

The prediction that populations subject to an Allee effect and dispersal must occupy a critical area to persist (Lewis & Kareiva 1993; Kot *et al.* 1996; Etienne *et al.* 2002; Soboleva *et al.* 2003) is important to our understanding of spatial population dynamics and for applications of ecological theory to conservation and invasion ecology, but has until now lacked empirical evidence. Our results confirm this prediction. In our study, the probability of persistence of gypsy moth populations from one year to the next was positively associated with population area in all 12 years of study, and we were able to separate the effect of area from attendant effects of location and density. Prior studies have reported the importance of density in gypsy moth persistence (Liebhold & Bascompte 2003; Whitmire & Tobin 2006), which was also an important factor in our study as persistence was positively associated with residual density in 10 of 12 years. Additionally, a mechanistic model specially built to understand Allee effects in gypsy moth was consistent with observed patterns supporting our overall conclusion that mate limitation in sparse populations leads to a critical area in this species.

Our inference regarding the importance of density and area in population persistence, as theoretically described by Lewis & Kareiva (1993), is strongly supported by our ability to control for other correlates of persistence, particularly factors known to vary geographically because of differences among regions (Tobin *et al.* 2007). In our models, spatial terms always explained a large proportion of the total deviance. These terms encompass both: (1) variation in habitat characteristics that are correlated with geography, as evidenced by the lack of support for models that included preferred host density, elevation and frost index and (2) higher probability of persistence nearer to the core invaded region, likely caused by rescue effects (Brown & Kodric-Brown 1977) as reflected in correlation of geographic terms with the Northwest-to-Southeast orientation of the invasion front.

An alternative explanation to our conclusion that this phenomenon is caused by a critical area effect is that because low-density populations tended to occupy smaller areas, these populations would also be vulnerable to extinction from demographic stochasticity. The following argument shows this explanation to be implausible. If demographic stochasticity from the classical sources of variance in fecundity and survival is to have caused the pattern we report here, then the probability of extinction must be non-negligible at moderate population sizes (i.e. > 10). However, reproductive rate is likely too large in this species for this to occur. For instance, consider a population with average individual reproductive output of Φ = 150 females distributed as a Poisson random variable. Assuming survival to be independent between individuals with a conservative probability π = 0.02 (Table S2), the combined offspring distribution will also be Poisson distributed with parameter πΦ = 3.0 (Johnson *et al.* 1993; Haccou *et al.* 2005). For small populations, we can therefore represent stochastic population growth by a density-independent branching process with a Poisson offspring distribution with mean 3. From the standard theory of discrete-time branching processes (Haccou *et al.* 2005), the theoretical probability of rapid extinction is then < 0.0002 for a population of three individuals, i.e. introduction of one fertilized female. It follows that mate limitation is the dominant cause of population extinction. We note, moreover, that our results agree with the model prediction that critical area should depend on initial population density (Appendix S3), in which low-density populations require larger initial areas to persist. In 6 of 12 years, we detected a significant negative interaction between population area and residual density on the probability of persistence that was driven by the very low-density populations. Thus, the relative effect of area on persistence was stronger in low-density populations (where residual density is negative) than in high-density populations. Such a pattern is characteristic of populations subject to an Allee effect, because the relative impact of the loss of individuals by dispersal on population growth rate is higher in low-density populations where the intrinsic growth rate is reduced (Robinet *et al.* 2008), and not an effect of demographic stochasticity.

The existence of critical areas in population persistence has important implications for conservation and invasive species management. Restoration strategies, for instance, usually entail that available habitat be large enough to limit the negative effects of demographic stochasticity and habitat fragmentation on long-term population persistence (Soulé 1987; Huxel & Hastings 1999). However, the chances of restoration program success could be further improved by considering the critical density threshold and the critical introduction area together (Courchamp *et al.* 2008). Indeed, if only a limited number of individuals are available for reintroduction, then the optimal reintroduction plan would distribute these individuals so that density is high enough to ensure a positive growth rate (Grevstad 1999), but over an initial area large enough to limit the loss of individuals by diffusion into the habitat. Such a strategy might also buffer effects of localized stochastic and catastrophic events (Courchamp *et al.* 2008). Conversely, the importance of critical areas could enhance control strategies against invasive species, particularly in eradication. The interplay between founder population density and the spatial extent over which the population exists could thus be exploited to better target control interventions. While the importance of critical area to policy making is clear, specific quantitative predictions may require improved estimates of population parameters. The sensitivity analysis of the mechanistic model of gypsy moth growth rate showed that the critical radius varied up to a factor of 10^2^ over the range of plausible parameter values (Figure S6), which implies a 10^4^-scale variation for the estimation of the critical area. Estimates of parameters in population growth models are similarly imprecise in many cases, especially for low-density populations (Coulson *et al.* 2001; Freckleton *et al.* 2006).

This study provides the first empirical demonstration of the importance of area in populations subject to Allee effects. We confirm that individual processes at the local scale such as reproduction, survival and dispersal can interact with spatial distribution to determine population persistence at large spatial scales. Our analyses were highly repeatable in time and the qualitative prediction of a critical area was conserved over the whole range of parameter values in the mechanistic model, providing robust indication that critical areas do not require stringent conditions to occur, and therefore are likely to be general properties of populations with strong Allee effects. This evidence for the link between critical density and spatial dynamics fills an important gap in our understanding of the ecological factors affecting the dynamics of small populations.

## SUPPORTING INFORMATION

**Appendix S1** Analysis of invasion data with GAMs.

**Appendix S2** Analysis of area change: detailed methods.

**Appendix S3** Model of per capita population growth rate.

**Figure S1** Distribution of super-polygon areas (a) and residual densities (b) over the 12 years of data.

**Figure S2** Comparison of models for population persistence. (a) AIC. (b) Proportion of deviance explained.

**Figure S3** Contour plots for the effect of latitude and longitude on population persistence and partial dependence plots for the effects of area and residual density in the 12 pairs of years.

**Figure S4** Relationship between population area and rate of area change for each year of analysis.

**Figure S5** Relationship between population density and female mating probability from simulated data and fitted mathematical function.

**Figure S6** Relationship between critical radius and density (a), survival rate (b), fecundity (c) and dispersal distance (d).

**Table S1** Number of seed polygons and super-polygons for each year of analysis.

**Table S2** Parameter values and range for the mechanistic model of growth rate.

As a service to our authors and readers, this journal provides supporting information supplied by the authors. Such materials are peer-reviewed and may be re-organized for online delivery, but are not copy-edited or typeset. Technical support issues arising from supporting information (other than missing files) should be addressed to the authors.
