# Supplementary material for: Critical patch size generated by Allee effect in gypsy moth, Lymantria dispar (L.)
Source: Ecol Lett. 2011 Feb;14(2):179–86. doi: 10.1111/j.1461-0248.2010.01569.x (PMC3064761; doi:10.1111/j.1461-0248.2010.01569.x)
Supplement: Supplementary file 1 [file ele0014-0179-SD1.doc]

**Text S1. Analysis of invasion data**

Three sets of spatial covariates thought to have the potential to influence persistence were considered in our models. These were elevation (from the Earth Resources Observation and Science Center, http://eros.usgs.gov), frost index (from the National Climatic Data Center, http://cdo.ncdc.noaa.gov), and preferred host density (Morin et al. 2005). These were estimated for each super-polygon by overlaying these map layers and calculating their area-weighted mean.

Generalized additive models always included a smoothing term on the latitude and longitude of the population centroid. Parametric covariates were population area (logarithm), residual density, the interaction of area and residual density, and habitat covariates (frost index, elevation, density of preferred host). Model fit was estimated by AIC values, and the model including the interaction of area and residual density had the lowest AIC in every year, and could not be distinguished from the more complicated model including frost index (Fig. S2a).

Because of missing data due to incomplete coverage of elevation and density of preferred host, models including these covariates had a different number of observations than other models; therefore they could not be directly compared with AIC values. We compared the amount of deviance explained between all models, and here again the model including the interaction of area and residual density performed as well as the most complex models (Fig. S2b).

Population area and residual density thus appeared as the best parametric predictors of population persistence, and the model including their interaction was selected as the best model:

*Persistence=log_10_(area)+residual_density +log_10_(area)*residual_density + smooth(lat,long)*

Results of this model for the 12 years of data are drawn in Fig. S3.

REFERENCES

Morin, R.S., Liebhold, A.M., Luzader, E.R., Lister, A.J., Gottschalk, K.W. & Twardus, D.B. (2005)*. Mapping host-species abundance of three major exotic forest pes*ts. Res. Pap. NE-726. U.S. Department of Agriculture, Forest Service, Northeastern Research Station, Newton Square, PA.
