# Supplementary material for: Critical patch size generated by Allee effect in gypsy moth, Lymantria dispar (L.)
Source: Ecol Lett. 2011 Feb;14(2):179–86. doi: 10.1111/j.1461-0248.2010.01569.x (PMC3064761; doi:10.1111/j.1461-0248.2010.01569.x)
Supplement: Supplementary file 2 [file ele0014-0179-SD2.doc]

**Text S2. Analysis of area change : detailed methods**

To calculate the change in population area, we analysed the intersection between superpolygons from each pair of consecutive years (year *t* and *t+1*) :

- If a polygon in year *t* did not intersect any polygon in year *t+1*, then the population was considered extinct, and its area in year *t+1* set to 0.
- If a polygon in year *t* intersected a polygon in year *t+1*, then the polygon from year *t* was considered as the ‘ancestor’ of the ‘descendant’ polygon in year *t+1*. The areas of the ancestor and descendant polygons were then compared to estimate whether the population was spreading (area of descendant larger than area of ancestor) or contracting (area of descendant smaller than area of ancestor).
- A descendant polygon could be assigned only one ancestor polygon. If a descendant polygon in year *t+1* had several potential ancestors, we assigned the ancestor polygon sharing the largest intersection with the descendant polygon as the single ancestor.
- An ancestor polygon could be assigned several descendant polygons. If an ancestor polygon in year *t* intersected several descendant polygons in year *t+1*, the area of the ancestor polygon was compared with the sum of the areas of the descendant polygons.

We graphically inspected the relationship between population area and change in area to determine if the change in population area from one year to the next was consistent with the model prediction of a critical area for persistence. Change in area was calculated as log10((area in year *t+1*/area in year *t*)+1). We rescaled the y-axis for better visualization and substracted 1 to the rescaled value to match the value of the ratio of areas. A ratio higher than 1 means that area in year *t+1* is larger than area in year *t*, thus the population is increasing. Figure S4 shows that the rate of area change increases with population area in all years, and that the critical area estimated from the analysis of population persistence is associated with a threshold in the rate of change.
