# Supplementary material for: Critical patch size generated by Allee effect in gypsy moth, Lymantria dispar (L.)
Source: Ecol Lett. 2011 Feb;14(2):179–86. doi: 10.1111/j.1461-0248.2010.01569.x (PMC3064761; doi:10.1111/j.1461-0248.2010.01569.x)
Supplement: Supplementary file 3 [file ele0014-0179-SD3.doc]

**Text S3. Model of multiplicative population growth rate**

For organisms with discrete generations, population growth from one generation to another is given by the relationship: *N_t+1_= λ.N_t_* To verify whether local life history processes could induce a critical area for population growth, we developed a mechanistic model of gypsy moth multiplicative population growth rate λ(*n*). Growth rate components are mating probability μ (a function of density *n*), fecundity *F*, juvenile survival *s*, and emigration rate *e*. All parameter values are given in Table S2.

(1)

Gypsy moths experience mate-finding failure at low densities, which induces a strong Allee effect. In North American gypsy moth populations, females are flightless and emit a pheromone to attract flying males for mating (Odell & Mastro 1980). The probability that a given male will detect a female depends on the distance between them, and the form of this relationship was estimated based on a mark-recapture experiment by (Robinet et al. 2008). Because our mechanistic model is not spatially explicit, we simulated data over a range of densities, and calculated the average female mating probability for each level of density following (Robinet et al. 2008). Based on these simulated data, we fitted the function (2) with a least-square method (Fig. S5). Mating probability μ depends on global population density *n* and is of the form:

(2)

Because of this Allee effect, we can define a threshold density, below which the population goes extinct and above which it grows exponentially. This threshold density is equal to *n_min_* in the absence of emigration, and increases with *e*.

For any given density above *n_min_*, one can derive the critical emigration rate *e_c_* below which the population grows and above which it goes extinct.

(3)

The actual value of the emigration rate is expected to depend on the interaction of population area, geometry and individual dispersal distance. We consider that the population is a disc, of radius *r* and individuals disperse on average to a distance δ.

If δ <*r,* the part of the population which is likely to emigrate is the outer band of width δ on the disc, but only a part of these individuals will actually disperse out of the circle (depending on their direction of movement).

On any point of this band (at a distance *x* from the center of the circle), the angle φ that defines the directions of movement that will make individuals disperse out of the populations is (from Al Kashi theorem):

(4)

The proportion of individuals in this exact point that will disperse out of the population is φ/2π. So the proportion of a small area around the point that is made of emigrants is :

(5)

And we integrate this over the possible values of *x* (between *r-* δ and *r* if δ <*r*), which gives equation (6a).

If δ *<r*:

(6a)

If *r<* δ *<2r*:

(6b)

If δ*>2r*, all individuals leave the population and *e*=1.

If the population is circular, then the emigration rate *e* depends on dispersal distance δ and population radius *r*. Equations (6a) and (6b) can be solved numerically to predict the critical radius *r_c_* in function of population density. For the default parameter values used in the model, the critical radius decreases exponentially when population density increases on a log/log scale (Fig. S6a, solid line). In dense populations, the critical radius is less than 50 meters, but it can go up to more than 5000 meters in low-density populations.

Sensitivity to model parameters

The default values for survival and fecundity were chosen following Robinet et al. (2008). Because our model of population growth rate considers only the female part of the population, the value of 150 eggs for the fecundity parameter corresponds to a total number of 300 eggs, assuming even sex-ratio. Gypsy moth dispersal is mostly wind-borne (dispersal of first instar larvae) and occurs essentially over short distances. In a field experiment, Mason and McManus (Mason & McManus 1981) recaptured 35.5 larvae/trap at a distance of 60 meters from the source, 13.5 larvae/trap at a distance of 120 meters, and 5.7 larvae/trap at a distance from 180 meters. These are consistent with a negative exponential distribution of dispersal distance with an average distance of 60 meters (default value).

Model sensitivity to the different life-history parameters (survival, fecundity and dispersal distance) was explored by analysing the influence of each parameter on the value of the critical radius, for a fixed density (5^e^-04). We solved the model numerically for a plausible range of values for each parameter (survival : 0.02-0.05, Campbell 1981, Robinet et al. 2008; fecundity : 100-600 eggs, i.e. 50 to 300 female eggs, Campbell 1967; dispersal distance δ : from 40 (median value of the exponential distribution) to 140 meters (90th percentile)) and we calculated the corresponding variation of the critical radius (Fig. S6b/c/d).

Fecundity and survival had negative relationships with critical radius. The effect of survival was almost linear on a log/log scale (Fig. S6b), while the effect of fecundity was more curvilinear on the same scale (Fig. S6c). Over the range of fecundity we considered, the value of critical radius varied from 38 to 90 meters, whereas it varied between 43 and 70 meters over the range of survival rate. Dispersal distance had a positive linear effect on critical radius, which varied between 120 and 470 meters over the range of possible dispersal distances (Fig. S6d). When the model parameters vary, there always is a critical population radius under which the population growth rate is lower than 1, therefore the qualitative prediction of the model is insensitive to the model parameters. In addition, we defined two alternative combinations of model parameters: an « optimistic » scenario, with values of survival and fecundity in the maximum range (Fig. S6a, dotted line), and a « pessimistic » scenario, with values of survival and fecundity in the minimum range (Fig. S6a, dashed line). The default values of the model are close to the optimistic scenario, which means that we are likely to underestimate the critical radius in comparison with real population dynamics of gypsy moths. The predictions from this model are thus conservative and robust to the uncertainty in parameter estimation.

REFERENCES

Campbell, R.W. (1967). The analysis of numerical change in gypsy moth populations. *For. Sci.*, 15, 1-33.

Campbell, R.W. (1981). Historical review. In: *The Gypsy Moth: Research Towards Integrative Pest Management* (eds. Doane, C.C. & McManus, M.L.). USDA Technical Bulletin 1584, U.S. Department of Agriculture, Forest Service, Northeastern Research Station, Newton Square, PA, pp. 65-86.

Mason, C.C. & McManus, M.L. (1981). Larval dispersal of the gypsy moth. In: *The Gypsy Moth: Research Towards Integrative Pest Management* (eds. Doane, C.C. & McManus, M.L.). USDA Technical Bulletin 1584, U.S. Department of Agriculture, Forest Service, Northeastern Research Station, Newton Square, PA, pp. 161-202.

Odell, T.M. & Mastro, V.C. (1980). Crepuscular activity of gypsy moth adults. *Env. Entomol.*, 9, 613–617.

Robinet, C., Lance, D.R., Thorpe, K.W., Onufrieva, K.S., Tobin, P.C. & Liebhold, A.M. (2008). Dispersion in time and space affect mating success and Allee effects in invading gypsy moth populations. *J. Anim. Ecol.*, 77, 966-973.
