# Supplementary figures and images for: Critical patch size generated by Allee effect in gypsy moth, Lymantria dispar (L.)
Source: Ecol Lett. 2011 Feb;14(2):179–86. doi: 10.1111/j.1461-0248.2010.01569.x (PMC3064761; doi:10.1111/j.1461-0248.2010.01569.x)

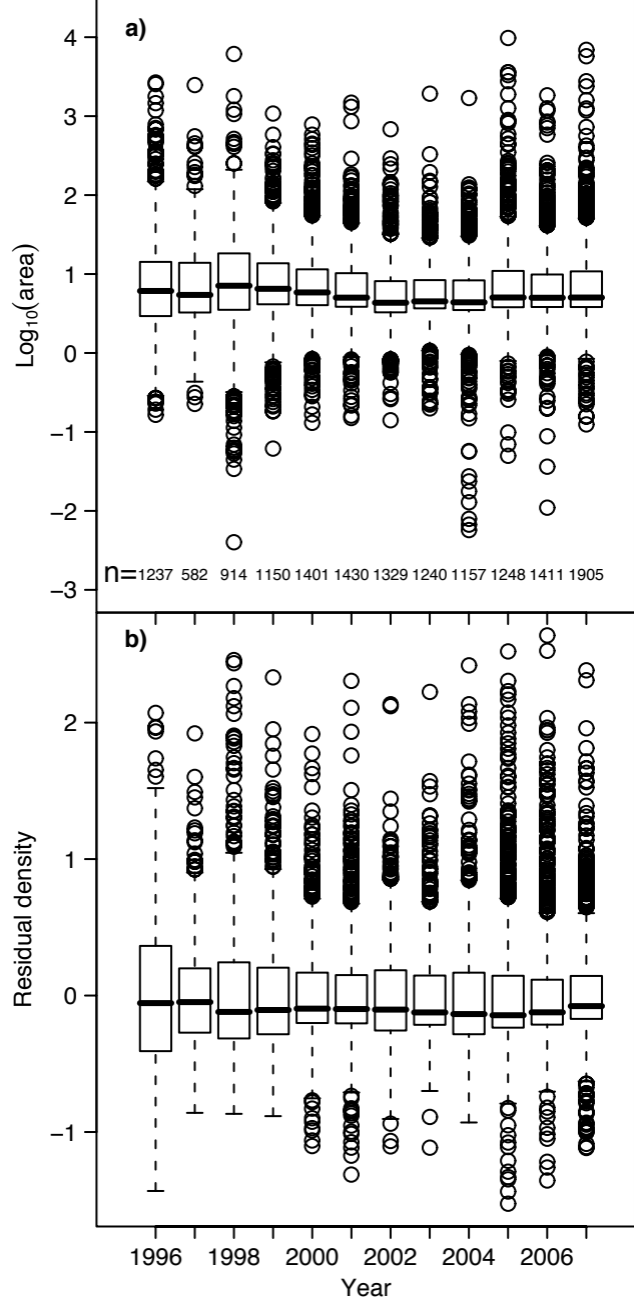

Supplement: Supplementary file 4 [file ele0014-0179-SD4.pdf]

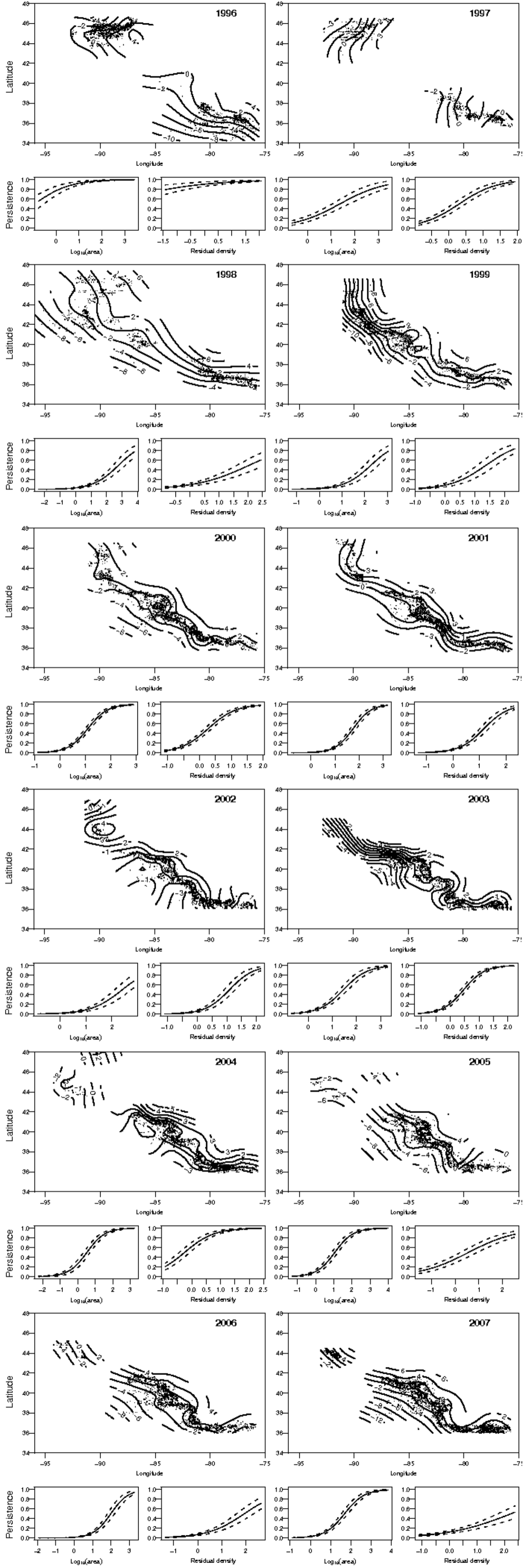

Supplement: Supplementary file 6 [file ele0014-0179-SD6.pdf]

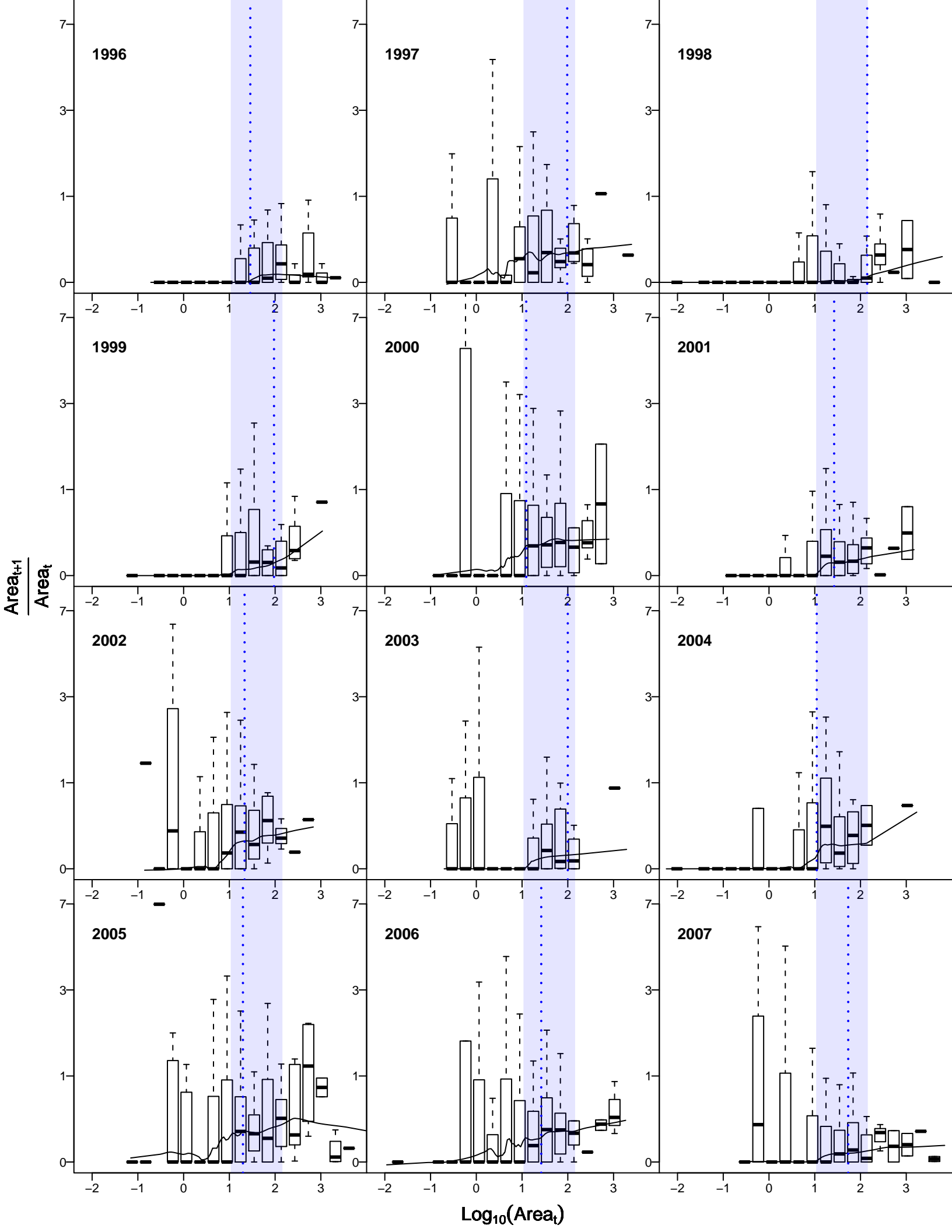

Supplement: Supplementary file 7 [file ele0014-0179-SD7.pdf]

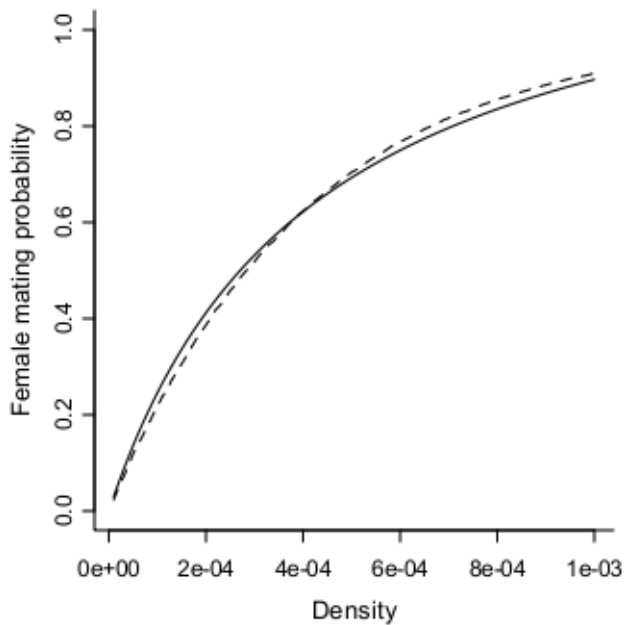

Supplement: Supplementary file 8 [file ele0014-0179-SD8.pdf]

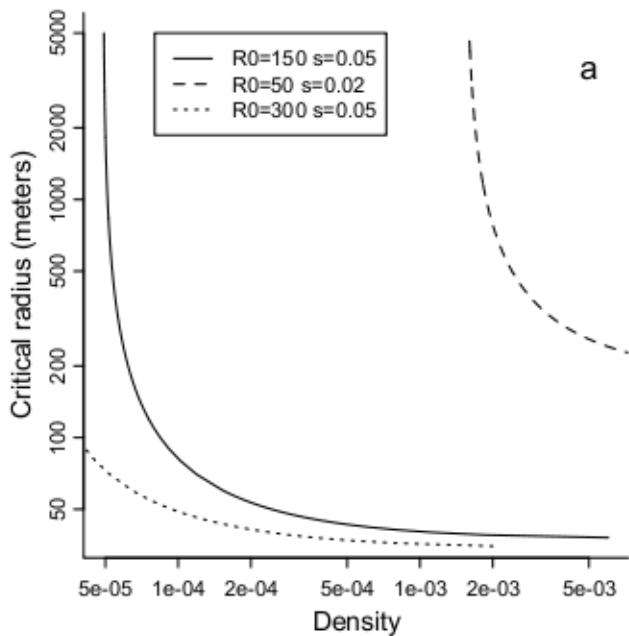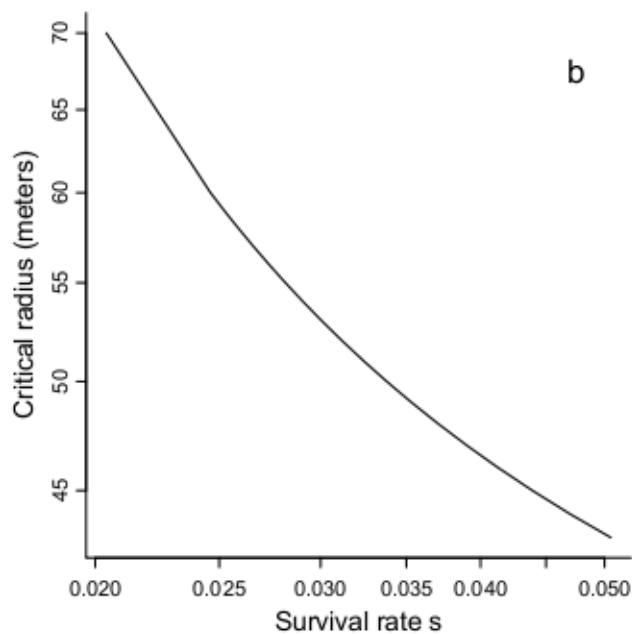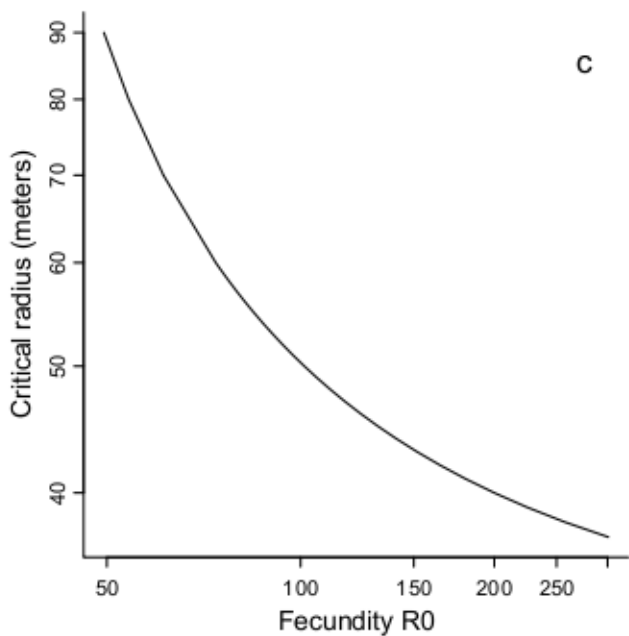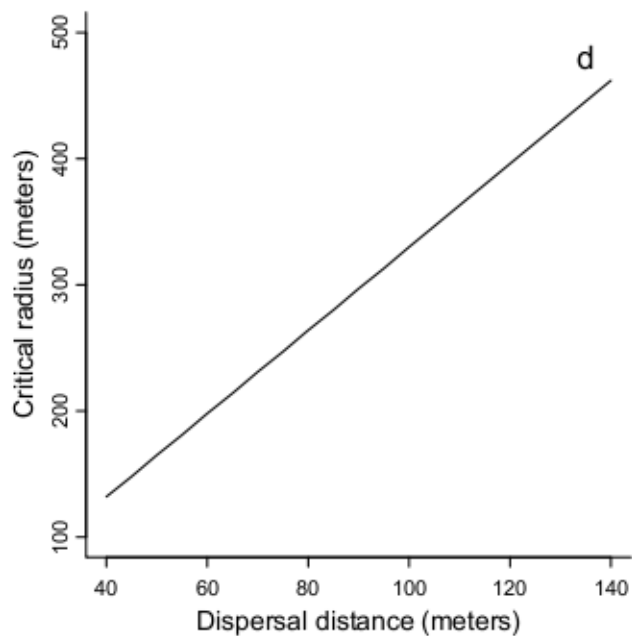

Supplement: Supplementary file 9 [file ele0014-0179-SD9.pdf]
