# Supplementary material for: Critical patch size generated by Allee effect in gypsy moth, Lymantria dispar (L.)
Source: Ecol Lett. 2011 Feb;14(2):179–86. doi: 10.1111/j.1461-0248.2010.01569.x (PMC3064761; doi:10.1111/j.1461-0248.2010.01569.x)
Supplement: Supplementary file 5 [file ele0014-0179-SD5.pdf]

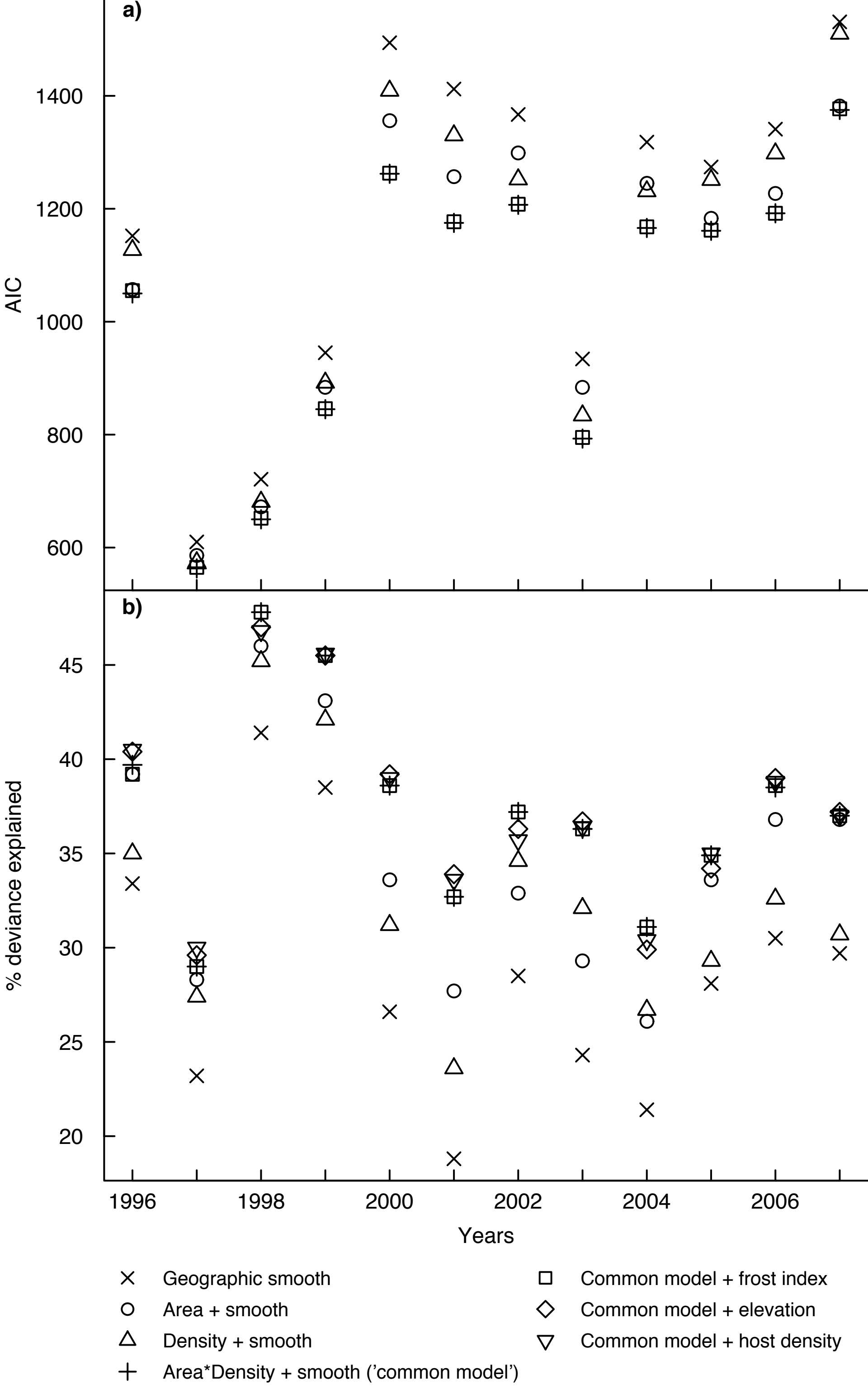

a)
b)
AIC
% deviance explained
Years
1996
1998
2000
2002
2004
2006
600
800
1000
1200
1400
20
25
30
35
40
45
Geographic smooth
Area + smooth
Density + smooth
Area*Density + smooth ('common model')
Common model + frost index
Common model + elevation
Common model + host density
