# Supplementary material for: Critical patch size generated by Allee effect in gypsy moth, Lymantria dispar (L.)
Source: Ecol Lett. 2011 Feb;14(2):179–86. doi: 10.1111/j.1461-0248.2010.01569.x (PMC3064761; doi:10.1111/j.1461-0248.2010.01569.x)
Supplement: Supplementary file 10 [file ele0014-0179-SD10.doc]

Table S1: Number of seed polygons and super-polygons for each year of study. Superpolygons that did not include any trap in the 2^nd^ year and very large super-polygons (more than 150 square kilometers) were removed for the final analysis.

| Year | Number of seed polygons | Initial number of super-polygons | Final number of super-polygons |
| --- | --- | --- | --- |
| 1996-1997 | 22517 | 1964 | 1203 |
| 1997-1998 | 12764 | 1450 | 572 |
| 1998-1999 | 19783 | 2097 | 896 |
| 1999-2000 | 18582 | 1957 | 1136 |
| 2000-2001 | 17433 | 1812 | 1383 |
| 2001-2002 | 21479 | 1802 | 1421 |
| 2002-2003 | 15275 | 1757 | 1325 |
| 2003-2004 | 19052 | 1853 | 1236 |
| 2004-2005 | 7965 | 1583 | 1156 |
| 2005-2006 | 10621 | 1539 | 1217 |
| 2006-2007 | 13518 | 1797 | 1393 |
| 2007-2008 | 13207 | 2353 | 1885 |
