# Supplementary material for: Critical patch size generated by Allee effect in gypsy moth, Lymantria dispar (L.)
Source: Ecol Lett. 2011 Feb;14(2):179–86. doi: 10.1111/j.1461-0248.2010.01569.x (PMC3064761; doi:10.1111/j.1461-0248.2010.01569.x)
Supplement: Supplementary file 11 [file ele0014-0179-SD11.pdf]

Table S2: Parameter values and range for the mechanistic model of growth rate

	s	F	α	β	δ
Default value	0.05	150 eggs	3048.8	2399.5	60 meters
Range	0.02-0.05	50-300	-	-	40-140
Source	Robinet et al. (2008)		Function fitted on mate detection probability from Robinet et al. 2008		Average dispersal distance for a negative exponential distribution (Mason and McManus 1981)
